# Letters on Syphilis, No 2

**Published:** 1852-01

**Authors:** 


*Letters on Syphilis, No* 2. By M. P. Ricord, of Paris. Translated by W. P. Lattimore, M. D., for the Med. Times.

*My Dear Confrere and Friend:*—I do not write a didactic essay; I would like so to do, but you know I have not at this time the power. I address to you familiar *Letters*, for which I claim all the privileges of the epistolary form; that is to say, liberty of style and spontaneity of thought. Thus, that which I did not say in the preceding letter, I will say, without formality, in this one; without a too rigid adherence to a plan, to a method, and to the other artifices of composition which are elsewhere so useful.

In order that my first letter should be complete in a hasty exposition of the attempts made in the way of experiment, I ought not to have omitted the mention of the efforts to inoculate syphilis from *man* to the *animals*. Either to get rid of the inconveniences which might result from the inoculation of man himself, or to resolve the curious problem of the transmission of syphilis to animals, Hunter and Turnbull had already attempted, but in vain, this inoculation from man to the animals. I had repeated all of these experiments, and had arrived at the same negative results. However, more recently, a young and laborious confrere, M. Auzias Turenne, has taken up these experiments; he has varied them; he has employed other methods than those which were known, and he thought that he had arrived at the experimental demonstration of the transmissibility of syphilis from man to certain of the animals. I then recommenced these experiments, and convinced myself anew, that syphilis was not strictly communicable to animals, and that the facts invoked by M. Auzias were illusive. M. Cullerier, at the "Hopital de L'Oursine," has studied this subject with much care, and has arrived at the same results with myself. My colleague, M. Vidal (de Cassis,) has, in his turn, experimented, and his conclusions, I believe, do not differ from mine.

Direct observation, then, experiment on the patient himself, were my only resources; to them alone did I resolve to address myself.

First of all, it was necessary to seek a certain source whence I could derive the cause, to the research of which I wished to direct all of my investigations. There must be no more trusting the statements of the patients; it was likewise necessary to avoid the objections justly brought against the experiments of Hunter and Harrison; to the facts reported by Bell, and to the experiments of Hernandez; and to do this, I first sought to well determine the state of the tissues from which I was about to borrow the reputed specific cause.

In fact, it could no longer suffice me, that (as Petronius formerly said,) a woman was reputed to be *tainted;* it was no longer sufficient to take, at random, a morbid secretion coming from the genital organs of a woman, and to make of it, according to the picturesque expression of Alexander Benedictus, *a venereal tincture* which should spread an uniform color over all the accidents resulting from it. No; the scientific tendencies of the spirits of my time, and the demands of my own reason, determined me to employ a method more decisive, and processes more rigorous.

I do not wish to lay too much blame upon the facility with which many have drawn their conclusions of the cause from the effect. But who can be otherwise than surprised that, in a question like this of venereal diseases.

where ignorance *or fraud*, in the words of Hunter, are such frequent causes of error; that, in a disease which, after all, and most invariably, is a flagrant proof of immorality, even the most judicious observers should as frequently place confidence in the dicta of patients, and invoke unceasingly the moral consideration of testimony.

Testimony! In such a matter, is there anything more deceptive; and particularly in respect to women? Let me cite to you two small examples, where you will see one of the most rigorous observers fall into the snare of a woman's testimony.

Babington wishes to destroy the law laid down by Hunter, that, when there is no pus nor a puriform secretion, the disease cannot be communicated; so that infection is not possible before the appearance of a gonorrhea, or after the cicatrization of a chancre. "This conclusion," says Babington, "is not without its dangers, as may be seen from the following facts, which are far from being rare.

"A married woman was seized with the ordinary symptoms of gonorrhea, which surprised her very much, as her husband was entirely exempt from disease. However, the husband, being questioned, confessed that he had had connection with a suspected woman about eight days before his wife discovered herself to be diseased; but he positively affirmed that he had had no discharge, nor any morbid sensation, and certainly he then showed no symptoms of the disease. At the end of four days, that is to say, about two weeks after the impure connection, and one week after he must have communicated the disease to his wife, a gonorrheal discharge manifested itself in him.

"A traveller exposed himself to the chances of a syphilitic infection, and three days afterward reached home. About four days subsequent to his arrival, his wife was attacked with gonorrhea; it was only ten days later that he was seized, for the first time, with a discharge and the other symptoms of gonorrhea." (John Hunter, *complete works;* notes by Babington.)

If, in the presence of such facts, Babington had sought, not to obtain more complete avowals, (there are avowals which women never make, even, as I have only too many occasions to see, under threatenings of the gravest dangers,) but to assure himself, by a serious examination, of the true state of things, he would assuredly have seen, in these cases, that the infecting cause was not in the genital organs of these candid husbands.

It was then no longer possible to think of founding any pathological truth whatever, in regard to syphilis, upon the truth of the testimony of patients; I had no more confidence in doctrines and facts, based upon the recitals of this nature.

It was necessary to dispel the mysteries of the closet, in order to expose to the broad light of day the cause which I sought.

This cause, where ought I first to seek it? At the very source; that is to say, in the genital organs of the woman; as well in their deepest folds, as in their external parts.

Fortune favored me. The "Hopital du Midi," at that time, received in its wards the unfortunates sent from the Dispensary.

Here, my dear friend, permit me to remind you, that previous to my entering the "Hopital du Midi," the manner of examining a woman consisted in seating her upon the edge of a chair, in separating the external organs of generation, and if no lesion of tissue was found there, every morbid secretion coming from above the vulva was referred to a gonorrheal discharge; at the vulva ring, my predecessors appeared to have placed for chancre, the pillars of Hercules.

I could not content myself, nor ought I so to have done, with this incomplete and superficial examination. We were not far removed from the epoch when M. Recamier had so fortunately exhumed the *speculum* from the surgical repertory. The fine applications which this celebrated practitioner made of the speculum to the diagnosis of diseases of the uterus are well known. But this precious instrument had not yet served for the diagnosis of syphilitic diseases; its employment, even in these cases, appeared, and was reputed to be, a contra-indication. I paid no attention to this widely circulated opinion; on the contrary, I used extensively the employment of the instrument upon all the women in the wards.

I do not know if posterity will partake of the opinion of one of my learned critics, who reduces to a very small sum all that it has been my fortune to accomplish in syphilopathy. However, my dear friend, when I recall the profound obscurities which enveloped the diagnosis of syphilitic diseases before the application of the speculum; when I compare the embarrassments of practitioners of that epoch in fixing their opinion, with the truly marvellous facility with which practitioners of our day can make a sure diagnosis; when I remember all the service which the speculum has already rendered to this department of practice, I believe that if my participation in the progress was limited to this, even then, the above opinion would be too rigid.

The employment of the speculum permits me to examine with the greatest care, all the surfaces venereally affected, and to verify with precision the state of the tissues which furnish the secretions.

These conditions being established, I thought to study all the modifications reputed as venereal, and compare them with other morbid secretions.

I commenced with gonorrhea.

You understand, my dear friend, that I ought to suppose my readers perfectly acquainted with the state of the question, concerning gonorrhea, at the time when I undertook my observations. Once more, I do not here write volumes of a complete history, but a simple and rapid exposition of facts peculiar to myself.

I sought to resolve, by experiment, this problem, already diversely resolved by the observations which you know.

Does gonorrhea recognize a specific cause? Hunter had learned that the pus of chancre, when inoculated, produces chancre. I said, if gonorrhea recognizes a specific cause, the muco-pus which it secretes, will, undoubtedly, when inoculated, produce phenomena similar to those produced by the inoculation of chancerous pus.

But in order to get accurate results; in order to isolate it from every complication, and to remove every cause of error, I ought, first of all, to inoculate the muco-pus coming from perfectly simple gonorrhea; to take this muco-pus from surfaces perfectly free from every ulceration; and you see how precious to me was the employment of the speculum; without it, those experiments were not possible.

Now, these first experiments, made in great numbers, and a long time continued, conducted me to this first fundamental result, which I arrange in the shape of a *proposition:*

**Every time that the muco-pus has been taken from a non-ulcerated mucous membrane, the results of inoculation have been negative.**

Every experimenter who has followed me in this path, has arrived at the same conclusion, and that, too, whatever may have been the period of the gonorrhea when the experiment was made.

Consequently, it was with great surprise that I read in your Journal the following passage, where M. Vidal, in his *Letters on Syphilitic Inoculations*, reproaches inoculation with having usually remained powerless, so far as gonorrhea is concerned: "In fact," says my learned colleague, "a distinguished pupil, M. Bigot, has attempted, under the eyes of M. Puche, Physician to the Hopital du Midi, sixty-eight inoculations with urethral muco-pus, and these sixty-eight inoculations have been without any kind of result!" I am astonished at the surprise of M. Vidal; these sixty-eight negative inoculations are entirely conformable to the facts which I had previously advanced; they confirm and corroborate my opinion on the rarity of *Syphilitic* gonorrhea; and when my contradictor asks you, "Do you believe, that among these sixty-eight gonorrheas, there were none with virus, none that bore the germ of Syphilis?" reply to him boldly, No; and precisely because the inoculation was negative.

A dialectician as skillful, a logician as rigid as M. Vidal, cannot avoid acknowledging that the results of experiment, on whatever subject exercised, are either positive or negative, and that scientifically, the negative results have no less value than the positive. The inoculation of the vaccine virus gives rise to no phenomenon in those subjects who have already had the small pox; is this negative result without importance and without consequence?

But, in kind, we will soon see how much of value and of force these negative results of inoculation have taken, when compared with the positive results of inoculation. I will mention, in passing, a first objection which will later find a complete refutation. Writers on syphilis have thought, with Hunter, that gonorrhea was a form of syphilis peculiar to the mucous membrane. I will limit myself, at present, to the remark that the experiments previously indicated, destroy this opinion from top to bottom; we shall hereafter see, that the pus of chancre, when applied to a mucous membrane, produces a chancre perfectly well.

From the experiments which have been indicated, I draw this *conclusion:* THE GONORRHEA WHOSE INOCULATED MUCO-PUS GIVES RISE TO NO RESULT, DOES NOT RECOGNIZE THE SYPHILITIC VIRUS FOR A CAUSE.

This conclusion, you are aware, has raised many and grave objections. But I fear you cannot, to-day, give me enough space to commence the exposition and the refutation. This will form, if you please, the subject of the third letter. Yours,

RICORD.

